# Association between biomarkers of inflammation and dyslipidemia in drug resistant tuberculosis in Uganda

**DOI:** 10.1186/s12944-024-02063-7

**Published:** 2024-03-01

**Authors:** Joseph Baruch Baluku, Robinah Nalwanga, Andrew Kazibwe, Ronald Olum, Edwin Nuwagira, Nathan Mugenyi, Frank Mulindwa, Felix Bongomin

**Affiliations:** 1 Kiruddu National Referral Hospital, Kampala, Uganda; 2 Faculty of Medicine, Mbarara University of Science and Technology, Mbarara, Uganda; 3 Makerere University Lung Institute, Kampala, Uganda; 4 MRC/UVRI Research Unit, Kampala, Uganda; 5 The AIDS Support Organisation, Kampala, Uganda; 6 School of Public Health, Makerere University College of Health Sciences, Kampala, Uganda; 7 United Health Services, Wilson Hospital, NY, USA; 8 Department of Medical Microbiology and Immunology, Faculty of Medicine, Gulu University, Gulu, Uganda

**Keywords:** Tuberculosis, Cholesterol, Inflammation, Dyslipidemia, Platelet, Mean corpuscular volume, Monocytes, Lymphocyte, HIV, HDL

## Abstract

**Background:**

Active tuberculosis (TB) significantly increases the risk of cardiovascular disease, but the underlying mechanisms remain unclear. This study aimed to investigate the association between inflammation biomarkers and dyslipidemia in patients with drug-resistant TB (DR-TB).

**Methods:**

This was a secondary analysis of data from a cross-sectional multi-center study in Uganda conducted 2021. Participants underwent anthropometric measurements and laboratory tests included a lipid profile, full haemogram and serology for HIV infection. Dyslipidemia was defined as total cholesterol > 5.0 mmol/l and/or low-density lipoprotein cholesterol > 4.14 mmol/l, and/or triglycerides (TG) ≥ 1.7 mmol/l, and/or high density lipoprotein cholesterol (HDL-c) < 1.03 mmol/l for men and < 1.29 mmol/l for women. Biomarkers of inflammation were leukocyte, neutrophil, lymphocyte, monocyte, and platelet counts, as well as neutrophil/lymphocyte (NLR), platelet/lymphocyte, and lymphocyte/monocyte (LMR) ratios, mean corpuscular volume (MCV), and the systemic immune inflammation index (SII) (neutrophil × platelet/lymphocyte). Modified Poisson Regression analysis was used for determining the association of the biomarkers and dyslipidemia.

**Results:**

Of 171 participants, 118 (69.0%) were co-infected with HIV. The prevalence of dyslipidemia was 70.2% (120/171) with low HDL-c (40.4%, 69/171) and hypertriglyceridemia (22.5%, 38/169) being the most common components. Patients with dyslipidemia had significantly higher lymphocyte (*P* = 0.008), monocyte (*P* < 0.001), and platelet counts (*P* = 0.014) in addition to a lower MCV (*P* < 0.001) than those without dyslipidemia. Further, patients with dyslipidemia had lower leucocyte (*P* < 0.001) and neutrophil (*P* = 0.001) counts, NLR (*P* = 0.008), LMR (*P* = 0.006), and SII (*P* = 0.049). The MCV was inversely associated with low HDL-C (adjusted prevalence ratio (aPR) = 0.97, 95% CI 0.94–0.99, *P* = 0.023) but was positively associated with hypertriglyceridemia (aPR = 1.04, 95% CI 1.00-1.08, *P* = 0.052).

**Conclusions:**

Individuals with dyslipidemia exhibited elevated lymphocyte, monocyte, and platelet counts compared to those without. However, only MCV demonstrated an independent association with specific components of dyslipidemia. There is need for further scientific inquiry into the potential impact of dyslipidemia on red cell morphology and a pro-thrombotic state among patients with TB.

## Background

Tuberculosis (TB) remains the leading infectious cause of mortality globally [1]. Moreover, individuals with active TB face a significantly high risk of major adverse cardiovascular events, estimated at 51% greater than those without TB [2]. This translates to increased incidence of ischemic stroke, peripheral artery disease, and myocardial infarction in patients with TB [3–6]. The underlying mechanisms of cardiovascular disease (CVD) remain unclear, but several factors contribute.

Patients with TB often exhibit a higher prevalence of traditional CVD risk factors, which could play a causal role in this increased incidence. A recent systematic review of CVD risk factors among patients with TB in Africa reported 26% smoking, 21% hazardous alcohol use, 14% hypertension, 7% diabetes, and 4% obesity [7]. Additionally, we have previously shown that 63% of patients with drug resistant TB (DR-TB) have dyslipidemia [8].

Beyond these traditional CVD risk factors, TB infection triggers a potent cell-mediated immune response, marked by an over-expression of pro-inflammatory cytokines (interleukin [IL]-1, IL-2, IL-6, interferon-gamma [IFNγ], tumor necrotic factor-alpha [TNFα]), alongside monocyte/macrophage, CD4 + T-helper 1, and T-helper 17 cell activation [9]. These immune responses are believed to contribute to atherogenesis, offering a potential mechanistic link to the observed increase in CVD risk among patients with TB [9].

Dyslipidemia (hyperlipidaemia and hypolipidaemia), characterized by elevated blood total cholesterol (TC), low-density lipoprotein cholesterol (LDL-c) and triglycerides (TG), and reduced high-density lipoprotein cholesterol (HDL-c) (hypolipidaemia) levels, presents a complex picture [10]. While canonically associated with CVD in the general population [11], elevated blood lipids in patients with TB have been linked to lower TB infection risk, less severe radiological disease, lower mycobacterial load, and lower all-cause mortality [12–14]. This association of hyperlipidemia and lower TB mortality further complicates the understanding of dyslipidemia and CVD-related mortality among patients with TB.

Several cell indices on the full blood count (FBC) serve as inflammation surrogate markers and CVD predictors. Elevated leucocyte, neutrophil, platelet counts, neutrophil to leucocyte ratio (NLR), platelet to lymphocyte ratio (PLR), mean corpuscular volume (MCV), red cell distribution width (RDW), and mean platelet volume (MPV) associate with CVD incidence and/or severity [15]. Among patients with TB, Wei et al. demonstrated that MPV and elevated monocyte percentage correlated with incident ischemic stroke [4]. However, the interplay between these inflammatory markers and dyslipidemia in TB remains poorly understood. A recent Taiwanese study suggested an inverse correlation between HDL-c and TC with the leucocyte count-to- NLR ratio, suggesting a potential link between HDL-c and lower systemic inflammation in patients with TB [14]. Further research is needed to fully understand this intricate relationship.

This study aimed to explore the association between dyslipidemia and inflammatory markers on the FBC in patients with TB, exploring potential mechanisms that could explain increased CVD risk observed in patients with TB. The study hypothesized that dyslipidemia and specific lipid abnormalities, would be associated with increased levels of inflammatory markers in people with TB, potentially contributing to the high prevalence of CVD this population.

## Materials and methods

### Study population and study design

This was an exploratory analysis of data from a cross-sectional study among adults ≥ 18 years with bacteriologically confirmed pulmonary DR-TB receiving treatment at four large treatment centers in Uganda. The detailed methodology of the primary study has been described elsewhere [8, 16]. Briefly, people were diagnosed with rifampicin-resistant TB using the *Mtb* Xpert/RIF assay. Baseline sputum samples were subsequently sent to the national TB reference laboratory for a full drug susceptibility test for resistance to isoniazid, rifampicin, fluroquinolones, and second-line injectable aminoglycosides. Consecutive eligible participants were enrolled during routine clinic visits and underwent anthropometric measurements (weight, height, waist, and hip circumference), blood pressure measurements, and blood draw for a non-fasting lipid profile, FBC, glycated hemoglobin (HbA1c), random blood glucose (RBG), and HIV testing. In this secondary analysis, participants who had data for both non-fasting lipid profile and FBC parameters were included.

### Data collection and study measurements

Participants provided informed consent and completed a questionnaire collecting sociodemographic information, smoking and alcohol use history, and any pre-existing medical conditions. A trained nurse performed anthropometric measurements, took blood pressure measurements and drew blood samples using standardized techniques.

Participants underwent anthropometric measurements of their weight, height and waist/hip ratio using a weighing scale (Seca 760®), stadiometer (Seca 213®) and tape measure respectively. The body mass index (BMI) was calculated using the formula; BMI = weight (kilograms)/height (in metres)^2^. Using a battery powered digital blood pressure (BP) machine (Omron®, Hem 7120), the BP was taken on two separate occasions, 20 min apart, at the DR-TB treatment centre. The average BP of the two measurements was considered as the participant’s BP. A study nurse drew 4 millilitres (mls) of blood which was tested for random blood glucose (RBG), glycated haemoglobin (HbA1c), non-fasting lipid profile, and a complete blood count. The HbA1c and blood lipids (TG, TC, LDL-c and HDL-c) were estimated using the Cobas® 6000 analyzer series (Roche Diagnostics, USA).

High body mass index (BMI) (weight [kilograms]/height^2^ [meters]) was defined as a BMI ≥ 25.0 kg/m^2^. Hyperglycemia was defined as HbA1c > 5.6% [17]. Central obesity was defined as waist circumference ≥ 102/88 cm and/or waist-hip ratio ≥ 0.90/0.85 for men/women, respectively [18].

### Study outcome

The primary outcome was dyslipidemia defined according to the National Cholesterol Education Program Adult Treatment Panel III (NCEP ATP III) guidelines [19]: TC > 5.0 mmol/L and/or LDL-c > 4.14 mmol/L and/or TG ≥ 1.7 mmol/L and/or HDL-c < 1.03 mmol/L for men and < 1.29 mmol/L for women. Low HDL-c was an HDL-c < 1.03 mmol/l for men and < 1.29 mmol/l for women and hypertriglyceridemia was TG ≥ 1.7 mmol/l. While hyperlipidemia was defined as elevated TC, LDL-c and TG, hypolipidemia was defined as low HDL-c using the aforementioned cut-offs. The LDL-c/HDL-c ratio was considered elevated if > 2.5 [20, 21].

The main exposure was inflammatory markers measured in the FBC known to predict CVD. Specific hemogram inflammatory markers analyzed were total leucocyte, neutrophil, lymphocyte, and monocyte counts; NLR, PLR, lymphocyte/monocyte ratio (LMR), MCV, RDW, and systemic immune-inflammation index (SII) (neutrophil × platelet/lymphocyte) [15, 22].

### Statistical analysis

Statistical analyses were performed using Stata version 17.0. Medians and the corresponding interquartile ranges were used to summarise continuous data while categorical variables were summarized as frequencies and percentages. Baseline characteristics were compared by dyslipidemia status using the Chi-square test (categorical variables) and the Kruskal-Wallis test (continuous variables). Because of the high prevalence of dyslipidemia, univariate and multivariate modified Poisson Regression models were employed to assess the association between inflammatory markers and dyslipidemia, as well as its most prevalent components, low HDL-c and hypertriglyceridemia. The multivariable models were adjusted for potential confounders: hyperglycemia, high BMI, HIV status, smoking history, and central obesity, selected based on their established relevance to CVD risk in existing literature. The data were tested for collinearity by calculating the variance inflation factor (VIF) of the variables in the model. Variables with a high VIF (that is, VIF > 10) were removed from the model. Specifically, the leucocyte and neutrophil counts, PLR and SII were removed from the multivariable model. Further evaluation of the correlation between the individual cell counts and the derived indices found weak correlation between these variables save for the NLR and PLR (Pearson correlation coefficient of 0.895) (Appendix 1). Hyperglycemia, high BMI, HIV status, smoking history, and central obesity were assessed whether they are potential modifiers by introducing interaction terms for each biomarker and no effect modification was found. Statistical significance was set at *P* < 0.05. A power analysis was not conducted for this secondary analysis as sample size requirements were estimated based on the primary study.

## Results

Out of 212 participants in the original study, 171 (80.7%) had data available for both lipid profiles and full blood counts.

### Characteristics of study participants

Among the 171 participants, 118 (69.0%) were co-infected with HIV (all on antiretroviral therapy), while 69 (40.4%) had central obesity and 60 (35.1%) reported a history of smoking. Notably, 15 (8.9%) had hyperglycemia and 14 (8.6%) had a high BMI (Table 1**)**. The mean (SD) RBG was 5.02 (1.54) mmol/l and was weakly positively correlated with the HbA1c (Pearson’s correlation coefficient = 0.11). The prevalence of dyslipidemia was 70.2% (95% CI 62.7–76.9). A total of 69 (40.4%) had low HDL-c (hypolipidemia), 38 (22.5%) had hypertriglyceridemia, 24 (16.9%) had elevated TC and, 11 (6.4%) had elevated LDL-c. As such, hypolipidaemia was the commonest form of dyslipidaemia. Notably, 158 (92.4%) had elevated LDL-c/HDL-c ratio. Patients with dyslipidemia had significantly higher lymphocyte (*P* = 0.008), monocyte (*P* < 0.001) and platelet counts (*P* = 0.014) in addition to a lower MCV (*P* < 0.001) than those without dyslipidemia. Further, patients with dyslipidemia had lower leucocyte (*P* < 0.001) and neutrophil (*P* = 0.001) counts, NLR (*P* = 0.008), LMR (*P* = 0.006) and SII (*P* = 0.049). There were no differences in age (*P* = 0.519) and sex (*P* = 0.358) distribution between the two groups.

**Table 1:** Characteristics of study participants and a comparison between patients with and without dyslipidemia

Characteristic	Overall (*n* = 171) n (%)	TB with Dyslipidemia (*n* = 120), n (%)	TB without Dyslipidemia (*n* = 51), n (%)	P-value
**DR-TB treatment site**				**< 0.001**
Mulago National Referral Hospital	61 (35.7)	31 (25.8)	30 (58.8)	
Lira Regional Referral Hospital	68 (39.8)	55 (45.8)	13 (25.5)	
Mbale Regional Referral Hospital	25 (14.6)	23 (19.2)	02 (3.9)	
Mbarara Regional Referral Hospital	17 (9.9)	11 (9.2)	06 (11.8)	
**Age, median (IQR), years**	37.0 (31.0, 46.0)	37.0 (30.0, 46.5)	38.5 (32.0, 46.0)	0.519
**Males**	126 (73.7)	86 (71.7)	40 (78.4)	0.358
**Rural residence**	104 (60.8)	78 (65.0)	26 (51.0)	0.086
**High BMI**	14 (8.6)	07 (14.6)	07 (6.1)	0.081
**Central Obesity**	69 (40.4)	44 (36.7)	25 (49.0)	0.132
**Positive HIV status**	118 (69.0)	80 (66.7)	38 (74.5)	0.310
**Any history of alcohol use**	75 (43.1)	55 (45.6)	20 (39.2)	0.425
**Any history of smoking**	60 (35.1)	41 (34.2)	19 (37.3)	0.699
**Duration of current TB treatment, median (IQR), months**	6.0 (5.0, 6.0)	6.0 (5.0, 6.0)	6.0 (5.0, 6.0)	0.533
**Antiretroviral therapy drugs (among people with HIV),** *** n*** ** = 73**				
Tenofovir	72 (96.0)	53 (98.1)	19 (100.0)	0.550
Lamivudine	72 (96.0)	53 (98.1)	19 (100.0)	0.550
Dolutegravir	61 (83.6)	47 (87.1)	14 (73.7)	0.177
Emitricitabine	1 (1.4)	1 (1.85)	0 (0.0)	0.550
Abacavir	1 (1.4)	1 (1.85)	0 (0.0)	0.550
Atazanavir/Ritonavir	1 (1.4)	1 (1.85)	0 (0.0)	0.550
Efavirenz	11 (15.1)	6 (11.1)	5 (26.3)	0.111
**Hemoglobin level, mean (SD), grams per deciliter**	14.2 (2.9)	13.8 (2.9)	14.95 (2.7)	**0.017**
**Blood lipids, median (IQR)**				
Total cholesterol, mmol/l	3.36 (2.7, 4.3)	3.2 (2.6, 5.2)	3.43 (3.1, 4.0)	0.516
High density lipoprotein cholesterol (HDL-c), mmol/l	1.25 (0.9, 1.7)	1.0 (0.8, 1.7)	1.6 (1.3, 1.7)	**< 0.001**
Triglycerides, mmol/l	1.20 (0.9, 1.6)	1.3(0.9, 02)	1.1 (0.8, 1.4)	**0.006**
Low density lipoprotein cholesterol (LDL-c), mmol/l	1.67 (1.2, 2.1)	1.7 (1.2, 2.4)	1.6 (1.2, 2.0)	0.245
LDL-c/HDL-c	1.42 (1.0, 1.9)	1.6 (1.2, 2.0)	1.1 (0.7, 1.4)	**< 0.001**
**Biomarkers of inflammation, median (IQR)**				
**Leucocyte count (X10** ^3^ **cells/microliter)**	5.71 (4.2, 8.9)	5.1 (4.0, 7.1)	7.7 (4.8, 13.7)	**< 0.001**
**Neutrophil count (X10** ^3^ **cells/microliter)**	3.42 (1.9, 7.8)	2.6 (1.7, 6.2)	6.6 (2.4, 11.4)	**0.001**
**Lymphocyte count (X10** ^3^ **cells/microliter)**	1.24 (0.3, 1.7)	1.4 (0.5, 1.9)	0.7 (0.1, 1.5)	**0.008**
**Monocyte count (X10** ^3^ **cells/microliter)**	0.30 (0.04, 0.6)	0.4 (0.1, 0.6)	0.04 (0.02, 0.2)	**< 0.001**
**Platelet count (X10** ^9^ **/litre)**	141.5 (81.0, 207.0)	161 (82.5, 219.0)	123.0 (59.0,162.0)	**0.014**
Mean corpuscular volume (femtolitres)	98.4 (87.8, 106.8)	94.3 (85.3, 104.3)	105.6 (92.4, 115.9)	**< 0.001**
NLR	13.1 (1.2, 49.9)	2.0 (1.1, 25.2)	15.7 (2.1, 82.2)	**0.008**
PLR	134.9 (75.8, 270.7)	130.4 (79.0, 208.6)	161.4 (63.0, 1050)	0.506
LMR	3.8 (2.3, 6.5)	3.6 (2.3, 5.0)	5.6 (3.0, 11.5)	**0.006**
RDW (%)	18.3 (14.9, 22.2)	17.3 (12.0, 21.6)	19.6 (17.9, 23.3)	0.050
SII	334.9 (170.5, 4548.0)	296.9 (161.4, 2033.1)	1960.8 (196.4, 8369.2)	**0.049**

BMI– Body mass index, NLR – neutrophil/lymphocyte ratio, PLR – platelet/lymphocyte ratio, LMR – lymphocyte/monocyte ratio, RDW – red cell distribution width, SII - systemic immune inflammation index (neutrophil × platelet/lymphocyte), DR-TB – drug resistant tuberculosis, XDR-TB – extensively drug resistant TB. Bolded p-values indicate statistically significant results

### Association between biomarkers of inflammation and dyslipidemia

In the multivariable analysis, no biomarker as independently associated with dyslipidemia on the overall (Table 2). Among the specific dyslipidemia components, the MCV was inversely associated with low HDL-C (adjusted prevalence ratio (aPR) = 0.97, 95% CI 0.94–0.99, *P =* 0.023) but was positively associated with hypertriglyceridemia (aPR = 1.04, 95% CI 1.00-1.08, *P* = 0.052). The association between the biomarkers of inflammation and the individual lipid abnormalities are shown in Table 2.

**Table 2:** Univariate and multivariate modified Poisson regression models for association between biomarkers of inflammation and dyslipidemia

Characteristic	cPR	95% CI	p-value	aPR	95% CI	P-value
	**Bivariable Analysis**			**Multivariable Analysis**		
**Modified poisson regression for associations with dyslipidemia**						
Lymphocyte count	1.15	0.93, 1.41	0.189	1.00	0.64, 1.57	0.990
Monocyte count	2.07	1.15, 3.73	**0.015**	1.08	0.24, 4.75	0.920
Platelet count	1.00	1.00, 1.00	0.189	1.00	0.99, 1.00	0.996
Red cell distribution width	0.98	0.94, 1.02	0.237	1.00	0.95, 1.06	0.871
Mean corpuscular volume	0.98	0.97, 0.99	**0.015**	0.98	0.96, 1.01	0.132
Lymphocyte Monocyte Ratio	0.98	0.95, 1.00	0.094	0.98	0.96, 1.01	0.295
Neutrophil Lymphocyte Ratio	1.00	0.99, 1.00	0.711	1.00	0.99, 1.00	0.893
Hyperglycemia (HbA1c > 5.6%)	1.36	0.75, 2.49	0.310	0.99	0.42, 2.31	0.973
High body mass index (≥ 25.0 kg/m^2^)	0.69	0.32, 1.49	0.345	0.46	0.11, 1.94	0.288
HIV infection	1.08	0.76, 1.55	0.663	1.30	0.80, 2.12	0.292
Any history of smoking tobacco	0.96	0.66, 1.40	0.883	1.07	0.65, 1.77	0.791
Central obesity	0.86	0.59, 1.24	0.411	0.80	0.50, 1.28	0.347
**Modified poisson regression for associations with low hdl-c**						
Lymphocyte count	0.88	0.66, 1,18	0.402	0.98	0.57, 1.68	0.936
Monocyte count	0.90	0.38, 2.10	0.801	1.29	0.22, 7.68	0.780
Platelet count	1.00	0.99, 1.00	0.334	1.00	0.99, 1.00	0.191
Red cell distribution width	1.05	1.01, 1.10	**0.017**	1.04	0.98, 1.10	0.211
Mean corpuscular volume	0.99	0.97, 1.01	0.206	0.97	0.94, 0.99	**0.023**
Lymphocyte Monocyte Ratio	0.99	0.97, 1.01	0.443	0.99	0.96, 1.02	0.487
Neutrophil Lymphocyte Ratio	1.00	0.99, 1.00	0.981	1.00	0.99, 1.00	0.510
Hyperglycemia (HbA1c > 5.6%)	1.26	0.54, 2.93	0.595	1.11	0.40, 3.07	0.846
High body mass index (≥ 25.0 kg/m^2^)	0.34	0.08, 1.39	0.134	0.65	0.15, 2.76	0.557
HIV infection	1.57	0.98, 2.52	0.063	1.73	0.96, 3.11	0.067
Any history of smoking tobacco	0.70	0.41, 1.19	0.191	0.71	0.37, 1.36	0.307
Central obesity	0.84	0.51, 1.37	0.486	0.78	0.44, 1.37	0.382
**Modified poisson regression model for associations with elevated tryglicerides**						
Lymphocyte count	0.88	0.59, 1.29	0.503	1.51	0.70, 3.27	0.293
Monocyte count	1.23	0.41, 3.71	0.705	0.88	0.07, 10.67	0.922
Platelet count	1.00	0.99, 1.00	0.596	1.00	0.99, 1.01	0.469
Red cell distribution width	1.01	0.94, 1.07	0.861	1.00	0.92, 1.09	0.966
Mean corpuscular volume	1.01	0.99, 1.03	0.438	1.04	1.00. 1.08	0.052
Lymphocyte Monocyte Ratio	0.97	0.92, 1.02	0.278	0.95	0.87, 1.03	0.231
Neutrophil Lymphocyte Ratio	1.00	1.00, 1.00	0.141	1.00	0.99, 1.00	0.286
Hyperglycemia (HbA1c > 5.6%)	1.72	0.67, 4.44	0.258	1.76	0.45, 6.86	0.418
High body mass index (≥ 25.0 kg/m^2^)	2.16	0.90, 5.20	0.086	1.63	0.33, 7.95	0.546
HIV infection	0.93	0.49, 1.78	0.835	0.87	0.39, 1.98	0.752
Any history of smoking tobacco	0.74	0.37, 1.49	0.400	0.73	0.30, 1.80	0.496
Central obesity	1.20	0.63, 2.28	0.572	0.78	0.35, 1.73	0.542

Systemic inflammatory index = neutrophil × platelet/lymphocyte; central obesity was defined as waist circumference of ≥ 102/88 cm and/or a waist-hip ratio of ≥ 0.90/0.85 in males/females; HDL-c – High density lipoprotein cholesterol, cPR – crude prevalence ratio, aPR – adjusted prevalence ratio, CI – confidence interval. Bolded p-values indicate statistically significant results

## Discussion

The aim of this study was to explore the association between dyslipidemia and inflammatory markers on the FBC in patients with TB. The study found a high prevalence of dyslipidemia among patients with TB (70%) that was predominantly due to low HDL-c and hypertriglyceridemia. Patients with dyslipidemia had elevated platelets, lymphocyte, and monocyte counts. The study also found that an increase in the MCV was positively associated with hypertriglyceridemia but inversely associated with low HDL-c. CVD is emerging as a significant cause of morbidity and mortality among patients with TB and TB survivors [2, 23]. While dyslipidemia and chronic inflammation are a cornerstone in atherogenesis [24], the association between markers of inflammation and dyslipidemia is not well characterized in TB. The study fills this crucial gap.

The high prevalence of dyslipidemia among patients with TB begs the question whether lipid lowering drugs have a role in reducing CVD risk among patients with TB. Compelling evidence suggests that statins not only reduce cholesterol levels (critical for *Mycobacterium tuberculosis* metabolism and cell wall integrity), thereby reducing active TB risk and severity, but also exert immune modulatory effects on monocytes, NK cells, and lymphocytes [25–29]. More studies are needed to establish whether statins prevent the initiation, progression and instability of atheroma plaques among people with TB; thereby reducing CVD risk. From the bivariate comparisons (Table 1), patients with dyslipidemia had higher lymphocyte and monocyte counts, suggesting that these cells could have a role in the interplay between dyslipidemia and inflammation. Few studies have reported the prevalence of dyslipidemia in patients with DR-TB. Similar to the current findings, Biranu and colleagues [30] found low HDL-c (65.5%) and hypertriglyceridemia (14.2%) to be the most prevalent forms of dyslipidemia in patients with multi-drug resistant TB. The mechanisms explaining the high prevalence are not apparent. However, in addition to inflammation and traditional CVD risk factors, it could be partly attributed to DR-TB treatment which alters gut microbiota to a phenotype that engenders elevated TC and LDL-c [31].

In the current study population, low HDL-c was the most predominant form of dyslipidemia. HDL plays a crucial role in the reverse cholesterol transport system, shuttling cholesterol from peripheral tissues back to the liver for excretion, thereby preventing the formation of atherosclerotic plaques [32]. Beyond this, HDL-c also boasts anti-oxidant, anti-thrombotic, and anti-inflammatory properties, including suppressing the expression of endothelial adhesion molecules and regulating the platelet activating factor, a potent activator of platelets, monocytes, and leukocytes [32]. In *Mycobacterium tuberculosis* infection, low HDL-c levels could exacerbate systemic inflammation, which high HDL-c has been shown to counter by suppressing tumor necrosis factor production [33]. However, the story of HDL-c is not as straightforward as it seems. While low levels are associated with increased CVD risk, indiscriminately raising HDL-c through pharmacotherapy has yielded disappointing results [34–36]. Emerging evidence suggests that specific sub-classes, particularly HDL3, have a more profound effect in protecting against CVD [37]. This shows a critical need for exploring drugs like fibrates, which selectively enhance HDL3 functionality, but their long-term association with mortality in patients with TB requires cautious consideration [32, 38].

The MCV is a measure of the average size of red blood cells and has been traditionally used for typing anemia [39]. Beyond its traditional role, emerging research links elevated MCV with endothelial dysfunction, increased severity of coronary artery disease, arterial stiffness, and a higher likelihood of major adverse cardiovascular events [40–45]. The current study delineates a nuanced relationship between MCV and dyslipidemia, revealing an inverse association with low HDL-c and a positive association with hypertriglyceridemia. Specifically, the study found that an elevated MCV corresponds to higher levels of both HDL-c and triglycerides. Though hypertriglyceridemia is a known culprit in CVD, it’s crucial to remember that high HDL-c, while often deemed protective, can be a double-edged sword. In fact, HDL-c levels exceeding 80 mg/100 ml have been associated with an increased risk of mortality, challenging the conventional understanding of its protective role in CVD [46].

The observation of an association between high MCV and elevated HDL-c levels, mirrors results from the National Health and Nutrition Examination Survey 2005–2006 [47] and studies involving overweight/obese individuals in Iran and non-anemic elderly populations [48, 49]. One plausible explanation is the role of plasma HDL-c as a cholesterol source for red blood cell membranes, leading to changes in red cell membrane cholesterol content and diameter. Such alterations can decrease red cell fluidity, stiffen the lipid shell, increase membrane density, and modify red cell morphology [50]. The potential role of the HDL-c on red cell membrane cholesterol is also supported by the positive association of the RDW, a measure of red cell size variability, and low HDL-c in the bivariate analysis [51]. The RDW has been associated with myocardial infarction, heart failure, stroke, atrial fibrillation, coronary artery disease, peripheral artery disease and hypertension [52]. The observed association between elevated RDW and HDL-c in coronary artery disease patients could be indicative of chronic inflammation and oxidative stress as well [53]. The results from this study collectively suggest that alterations in HDL-c levels can significantly impact red cell membrane characteristics, potentially affecting blood rheology and predisposing individuals to CVD. Although patients with dyslipidemia had significantly elevated platelet counts than those without, further investigation is needed to determine if this, along with red blood cell membrane alterations, contributes to a pro-thrombotic state in this population.

### Study strengths and weaknesses

The merits of the study lie in the multi-center nature of the study and the use of readily available markers of inflammation. Despite its valuable insights, the current study has limitations that warrant consideration. Due to its snapshot nature, changes in blood lipids and inflammation markers over time could not be assessed, particularly regarding the influence of TB treatment. This limits the understanding of any dynamic interplay between these factors and their long-term implications. Although some studies suggest HDL-c and total cholesterol remain stable during TB treatment [54], the participants in the current study had been on TB treatment for a median of 6 months. This raises the possibility that baseline lipid and inflammation marker levels may have differed from those at study entry, potentially affecting the observations. Longitudinal studies would be better suited to capture these dynamics over time. Another concern is the generalizability of the results. The research focused exclusively on patients with DR-TB, raising questions about the applicability of the findings to patients with drug-susceptible TB. While the prevalence of traditional CVD risk factors is reportedly similar in both DR-TB and drug-susceptible TB populations [7], it remains unclear whether the interplay between lipids and biomarkers of inflammation would differ according to TB drug resistance status. Genetically diverse strains of *Mycobacterium tuberculosis* exhibit varied propensity to metabolize host cholesterol but the effect on overall lipid levels of the host is unclear [55]. This aspect warrants further investigation. While the study evaluated readily available markers on a full haemogram known to predict CVD, we did not investigate the interaction of blood lipids with established inflammatory biomarkers such as C-reactive protein, erythrocyte sedimentation rate, interleukin-6, and tumor necrosis factor. These data were not collected from the study population. Future investigations could explore the potential synergistic effects of these inflammatory markers with blood lipids on CVD risk.

## Conclusion

In conclusion, the study highlights a significant prevalence of dyslipidemia among patients with DR-TB, primarily characterized by low HDL-c levels and hypertriglyceridemia. Notably, the results showed elevated monocytes, platelets and lymphocytes among patients with dyslipidemia as well as an association between the MCV with elevated HDL-c and hypertriglyceridemia. These findings underscore the intricate interplay between lipid metabolism and hematological changes in patients with TB. Crucially, the associations with MCV suggest that dyslipidemia may influence red blood cell morphology, potentially leading to alterations in blood rheology. This observation, coupled with the noted changes in platelet count, points towards a possible pro-thrombotic state in patients with TB with dyslipidemia. Such hematological alterations could have significant implications for the cardiovascular health of these patients.

## Appendix 1

**Table:** Correlation between cell counts and derived cell ratios

	Neutrophil count	NLR	Lymphocyte count	PLR	Platelet count	Monocyte count	LMR
**Neutrophil count**	1.000						
**NLR**	0.244	1.000					
**Lymphocyte count**	-0.301	-0.483	1.000				
**PLR**	0.133	0.895	-0.433	1.000			
**Platelet count**	-0.101	-0.046	0.294	0.070	1.000		
**Monocyte count**	-0.394	-0.318	0.652	-0.286	0.4168	1.000	
**LMR**	0.331	-0.113	0.192	-0.113	-0.213	-0.273	1.000

NLR: Neutrophil-Lymphocyte ratio; PLR: Platelet-Lymphocyte ratio; LMR: Lymphocyte-Monocyte ratio

## Data Availability

The datasets used and/or analysed during the current study are available from the corresponding author on reasonable request.

## Abbreviations

CVD: cardiovascular disease
LDL-c: low density lipoprotein cholesterol
HDL-c: high density lipoprotein cholesterol
HbA1c: glycated haemoglobin
BMI: body mass index
TB: tuberculosis
DRTB: drug resistant tuberculosis
IQR: interquartile range
RDW: red cell distribution width
NLR: neutrophil lymphocyte ratio
MCV: Mean corpuscular volume
MPV: mean platelet volume
SII: systemic inflammation index
LMR: lymphocyte/monocyte ratio
